# Human CD8+ T Cells Release Extracellular Traps Co-Localized With Cytotoxic Vesicles That Are Associated With Lesion Progression and Severity in Human Leishmaniasis

**DOI:** 10.3389/fimmu.2020.594581

**Published:** 2020-10-08

**Authors:** Carolina Cattoni Koh, Amanda B. Wardini, Millene Vieira, Livia S. A. Passos, Patrícia Massara Martinelli, Eula Graciele A. Neves, Lis Riberido do Vale Antonelli, Daniela Faria Barbosa, Teresiama Velikkakam, Eduardo Gutseit, Gustavo B. Menezes, Rodolfo Cordeiro Giunchetti, Paulo Roberto Lima Machado, Edgar M. Carvalho, Kenneth J. Gollob, Walderez Ornelas Dutra

**Affiliations:** ^1^ Laboratório de Biologia das Interações Celulares, Departamento de Morfologia, Instituto de Ciências Biológicas, Universidade Federal de Minas Gerais, Belo Horizonte, Brazil; ^2^ Laboratório Profa. Conceição Machado, Departamento de Morfologia, Instituto de Ciências Biológicas, Universidade Federal de Minas Gerais, Belo Horizonte, Brazil; ^3^ Laboratório de Biologia e Imunologia de Doenças Infecciosas e Parasitárias, Instituto René Rachou, FIOCRUZ-MG, Belo Horizonte, Brazil; ^4^ Center for Gastrointestinal Biology, Departamento de Morfologia, Instituto de Ciências Biológicas, Universidade Federal de Minas Gerais, Belo Horizonte, Brazil; ^5^ Serviço de Imunologia, Universidade Federal da Bahia, Salvador, Brazil; ^6^ Instituto Nacional de Ciência e Tecnologia em Doenças Tropicais, INCT-DT, Salvador, Brazil; ^7^ International Research Center, A.C. Camargo Cancer Center, São Paulo, Brazil

**Keywords:** extracellular traps, CD8+ T cells, etosis, cytotoxicity, pathology, leishmaniasis

## Abstract

Cell death plays a fundamental role in mounting protective and pathogenic immunity. Etosis is a cell death mechanism defined by the release of extracellular traps (ETs), which can foster inflammation and exert microbicidal activity. While etosis is often associated with innate cells, recent studies showed that B cells and CD4+ T cells can release ETs. Here we investigate whether CD8+ T cells can also release ETs, which might be related to cytotoxicity and tissue pathology. To these ends, we first employed an in vitro system stimulating human CD8+ T cells isolated from healthy volunteers with anti-CD3/anti-CD28. Using time-frame video, confocal and electron microscopy, we demonstrate that human CD8+ T cells release ETs upon stimulation (herein LETs – lymphocyte extracellular traps), which display unique morphology and functional characteristics. CD8+ T cell-derived LETs form long strands that co-localize with CD107a, a marker of vesicles containing cytotoxic granules. In addition, these structures connect the LET-releasing cell to other neighboring cells, often resulting in cell death. After demonstrating the release of LETs by human CD8+ T cells in vitro, we went on to study the occurrence of CD8-derived LETs in a human disease setting. Thus, we evaluated the occurrence of CD8-derived LETs in lesions from patients with human tegumentary leishmaniasis, where CD8+ T cells play a key role in mediating pathology. In addition, we evaluated the association of these structures with the intensity of the inflammatory infiltrate in early and late cutaneous, as well as in mucosal leishmaniasis lesions. We demonstrated that progression and severity of debilitating and mutilating forms of human tegumentary leishmaniasis are associated with the frequency of CD8+ T cells in etosis, as well as the occurrence of CD8-derived LETs carrying CD107a+ vesicles in the lesions. We propose that CD8+ T cell derived LETs may serve as a tool for delivering cytotoxic vesicles to distant target cells, providing insights into mechanisms of CD8+ T cell mediated pathology.

## Introduction

T cell activation and function is dependent on mechanisms that involve direct cell-cell interaction, as well as autocrine, paracrine and endocrine responses to soluble factors produced by a variety of cells. While CD4+ T cells are major orchestrators of the immune response by producing cytokines and mediating B cell activation, CD8+ T cells are classically associated with cytotoxic functions. Cytotoxicity involves the coordinated activity of a series of enzymes, which are delivered by lytic granules containing lysosomal-associated membrane proteins (LAMP), including CD107a (LAMP-1) (1). The mechanisms of cytotoxic granule delivery described to date involve an intimate proximity between the cytotoxic cell and its target (2–4). This delivery triggers cell death via both apoptotic-mediated mechanisms, and those independent on apoptosis (1, 3, 5).

Apoptosis, necrosis and etosis are distinct cell death mechanisms that occur under physiological and pathological circumstances and are critical for maintaining cell and tissue homeostasis. While apoptosis is the result of a misbalance between pro- and anti-apoptotic factors and caspase activation, leading to biochemical and morphological changes such as membrane asymmetry, chromatin fragmentation, and release of intracellular contents in apoptotic bodies (6–8), necrosis is characterized by massive disintegration of organelles, accumulation of intracellular water, and subsequent release of intracellular content in a disorderly manner (9). Etosis is an event in which cells release extracellular nets or traps (ETs, hence the name etosis), usually leading to cell death in a mechanism distinct from apoptosis and necrosis (10, 11). ETs are mainly composed of DNA and histones, but cytoskeletal proteins may also be present (12). Innate immune cells release ETs in response to bacterial and parasite components, as well as following in vitro stimulation with PMA and LPS (10, 13–17).

Neutrophil derived ETs play an important role in the control of infections, since they are capable of physically retaining and eliminating pathogens (18–20). One such example is the protozoan parasite *Leishmania*, which is susceptible to neutrophil-derived ETs (21, 22). Despite exerting a potentially protective role against pathogens, ETs can also amplify inflammatory responses, which may be detrimental if not controlled (23). Human infection with *Leishmania* leads to a spectrum of debilitating, mutilating and potentially deadly diseases. The cutaneous and mucosal manifestations of tegumentary leishmaniasis have been associated with exacerbated inflammatory and cytotoxic responses (24, 25), in which CD8+ T cells play a fundamental role (26–28).

While etosis is mostly related to innate immune cells, it was recently shown that B cells can release ETs in response to CpG stimulation (29), and that CD4+ T cells can also release ETs (16, 30). Thus, we hypothesized that CD8+ T cells can also release ETs, and that these ETs might be associated with CD8-mediated cytotoxic function and play a role in tissue pathology in human diseases. Thus, we performed a series of studies to determine if CD8+ T cells can release ETs and if they are associated with severity in human leishmaniasis, a devastating disease that affects millions worldwide and is a well-established example of CD8-mediated pathology. Here we unequivocally demonstrate that human CD8+ T cells release ETs that display a unique morphology upon activation (herein referred to as LETs - lymphocyte-derived ETs). Importantly, we show that CD8-derived LETs connect the LET-releasing cell with target cells, and co-localize with CD107a, a marker of cytotoxic granules. In addition, we show that progression and severity of human tegumentary leishmaniasis is associated with the release of CD107a+ LETs by CD8+ T cells. Our findings suggest that delivery of CD107a+ vesicles by CD8-derived LETs may provide an alternative mechanism of CD8-mediated cytotoxicity, with implications for disease pathology, and on the design of new therapeutic interventions.

## Patients, Materials, and Methods

### Patients With Leishmaniasis and Healthy Donors

Fifteen healthy volunteers were enrolled in this study for the various experiments of T cell activation to detect etosis, apoptosis, cell activation, as well as cytotoxic molecule and cytokine expression. All in vitro experiments were performed using cells from healthy donors.

A total of twenty-one leishmaniasis patients from the endemic area of Corte de Pedra, state of Bahia, Brazil were also enrolled in the study. We evaluated the presence of extracellular DNA (LETs), as well as determined the frequency of CD8+CD107+ LETs in lesions from cutaneous and mucosal leishmaniasis patients. Patients’ medical care, evaluation, and characterization were under the responsibility of Drs EC e PM. Diagnosis for leishmaniasis was performed based on clinical and laboratorial criteria. Detection of suggestive skin or mucosal lesions was associated to positive skin Montenegro test, parasite isolation and/or histopathological analysis to confirm diagnosis of CL or ML. For all CL and ML cases parasite species were typed to confirm that disease was due to *L. braziliensis* infection. Cutaneous patients enrolled in this study (total n=14) presented with a single ulcerated lesion and had not been previously diagnosed or treated for leishmaniasis. CL patients were classified as early-stage cutaneous leishmaniasis (Early-CL – approximately 15 days of illness, non-ulcerated palpular lesion) or late-stage cutaneous leishmaniasis (Late-CL – approximately 60 days of illness, classical ulcerated lesion), as previously established by us (26, 31). Mucosal patients (total n=7) presented with nasal lesions and, at the time of biopsy collection, and did not display concomitant cutaneous disease. Skin and mucosal biopsies were obtained from the edges of the active lesions with a 4-mm punch or scalpel, after the application of local anesthetic, processed and stored as previously done by us (24, 26). Samples were collected before treatment, which was offered to all patients as needed, despite their enrollment in this project. The ethics committees of the Federal Universities of Bahia and Minas Gerais approved all the procedures involved in this study and all individuals signed an informed consent.

### Histological and Immunofluorescence Staining in Tissues

Biopsies were used to obtain sections of 4 to 5 µm were placed on polarized slides and fixed for 10 min in acetone at -20°C or 15 min in 4% paraformaldehyde at room temperature. Slides were incubated with phosphate buffered saline (PBS) for 15 min and stained with hematoxylin-eosin or submitted to immunofluorescence. Immunofluorescence reactions were done using fluorescein isothiocyanate (FITC) - conjugated to monoclonal antibodies directed to surface receptors (CD4, CD8, CD68, CD107, Biolegend, San Diego, CA, USA) or anti-histone (Invitrogen, San Diego, CA, USA). Sections were incubated with antibody mixtures overnight at 4°C, followed by extensive wash with PBS. Samples were permeabilized for 1 h with 0.01% Triton X-100 prior to anti-histone and anti-CD107 stainings. Preparations stained with anti-histone were subsequently incubated with a Donkey anti-mouse IgG (H + L) secondary antibody labeled with Alexa Fluor 488 for 1 h. Finally, all slides were stained with 4’,6’-diamidino-2-phenylindole (DAPI, Molecular Probes, Eugene, OR, USA) and mounted using Vectashield® (Burlingame, CA, USA) mounting medium. Slides were kept at 4°C, protected from light, until they were acquired in a laser scanning confocal microscope (Zeiss 5 Live or Zeiss LSM 880), using an oil immersion objective (40×, 1.3 Oil). A water-cooled argon UV laser (488 nm) or a krypton/argon laser was used to excite the preparation. Images were analyzed using ImageJ 1.48v software. Isotype controls were used to confirm the lack of nonspecific staining. Frequency of cell subpopulations were determined by histological staining (mononuclear and polymorphonuclear) or by immunofluorescence (using fluorescence labeled anti-CD4, anti-CD8, anti-CD68, anti-CD107 monoclonal antibodies), and were performed by counting the total number of cells in a minimum of three acquired fields/slide, which was used to calculate the mean number of positive cells/section for each patient. Intensity of the inflammatory infiltrate was determined by counting the number of DAPI+ cells within the connective tissue and calculating the number of cells/field.

### Separation, Plating, and Culture of Human Peripheral Blood Mononuclear Cells (PBMC)

Purification of peripheral blood mononuclear cells (PBMC) was performed as previously done by us (32). Briefly, heparinized blood was applied over a Ficoll-Hypaque (GE Healthcare Life Sciences) gradient, centrifuged at 600*g* for 40 min, at room temperature, and PBMC were collected at the interface between the plasma and Ficoll. Cells were washed 3 times by centrifugation with PBS and resuspended in RPMI supplemented with antibiotic (penicillin 200 U/ml and streptomycin 0.1 mg/ml; Sigma, St. Louis, MO, USA) and l-glutamine (1 mM; Sigma, St. Louis, MO, USA) to a concentration of 10^7^ cells/ml. Lymphocyte-enriched fraction (LEF) was obtained by collecting non-adherent cells, after a 1-h incubation at 37°C in 6 flat-bottom well plates, using an initial concentration of 4 × 10^6^ cells/well. LEF were placed in 13-mm glass coverslips pretreated with poly-L-lysine (0.01%), at a concentration of 5 × 10^5^ cells/coverslip for electron microscopy, or 1 × 10^5^ cells/coverslip for immunofluorescence. LEF were stimulated with anti-CD3 and anti-CD28 monoclonal antibodies (2 μg and 1 μg/ml, respectively – Biolegend, San Diego, CA, USA), or incubated with media only for 24 h. In addition, cells were induced to lysis (1 h at -80°C) or necrosis (1 h at 56°C), as positive controls of DNA release and cell death. Samples were also submitted to treatment with DNAse (Ambion™ DNase I RNase-free, Invitrogen, Carlsbad, CA, USA) using 2 U/µl, by incubation at 37°C for 6 h.

LEF were also incubated in a polypropylene tube under the same conditions described above and used for flow cytometry analysis to determine cell death and expression of TNF. Supernatant from stimulated cultures, treated or not with DNAse, were collected for DNA and lactate dehydrogenase (LDH) measurement.

### Purification and Stimulation of Sorted CD8+ and CD4+ T Lymphocytes

CD8+ and CD4+ T lymphocytes were purified from the LEF (obtained as described above), using a FACS Aria II flow cytometer (Becton Dickinson, San Jose, CA, USA). Briefly, LEF were incubated with anti-CD8 and anti-CD4 monoclonal antibodies for 30 min at 4°C, washed twice with PBS, and resuspended at the concentration of 1 × 10^6^ cells/ml. Cells were submitted to the sorting procedures, as previously done (33), and yielded a purity of over 95%, as determined by flow cytometry. For some assays (as indicated in the text), purified lymphocytes were labeled with 5 mM carboxyfluorescein succinimidyl ester (CFSE, Sigma, St. Louis, MO, USA) by incubation for 10 min at room temperature, washed with PBS, plated in coverslips and stimulated or not with anti-CD3 and anti-CD28, as described above, for 24 h. Preparations were then processed for immunofluorescence and electron microscopy analysis.

### Immunofluorescence Reactions in Cell Monolayers and Confocal Microscopy Analysis

For immunofluorescence labeling, coverslips containing stimulated and non-stimulated cells were fixed with 2% paraformaldehyde by incubation for 15 min at room temperature. Cells were permeabilized with 0.01% Triton X-100 for 3 min, and the coverslips were incubated with a blocking solution of 1% bovine serum albumin (BSA) + 0.1% tween 20 for 1 h. Coverslips were incubated overnight with monoclonal antibodies directed to the different molecules (anti-histone, anti-CD8-Pe-Cy5, anti-CD107a-FITC – Biolegend, San Diego, CA, USA). Subsequently, samples were washed with PBS exhaustively. For the anti-histone staining, samples were incubated with the Donkey anti-mouse IgG (H + L) secondary antibody labeled with Alexa Fluor 488 for 1 h, and then washed with PBS. To stain with Alexa Fluor 594-phalloidin, cells were permeabilized, incubated for 20 min, and washed.

Finally, DNA of all samples was stained with DAPI (1:500) and/or with propidium iodide (PI) for 15 min at room temperature. Slides were extensively washed with PBS, coverslips were mounted with Vectashield® (Abcam, San Francisco, CA, USA), and remained at 4°C protected from light until acquisition in a laser confocal microscope.

Imaging was performed with a Zeiss 5 LIVE or Zeiss LSM 880 (software ZEN 2009), using an oil immersion objective (40×, 1.3 Oil). A water-cooled argon UV laser (488 nm) or a krypton/argon laser were used to excite the preparation (through its 363 nm line, 488 nm line or 568 nm line), and light emitted was selected with band pass filters (515-565 for FITC, 445/50 for DAPI and 575-640 for PI). Images were analyzed using ImageJ 1.48v software. ETs were analyzed by observing extracellular structures stained with DAPI and/or PI and/or histone.

To quantify the frequency of ETs and number of cells releasing ETs in each preparation, we counted the number of extracellular structures that were positive for DAPI and/or PI, as well as the number of cells that were associated with these structures, respectively. Results were expressed as ratio of the stimulated culture over the media control.

### Transmission and Scanning Electron Microscopy (EM)

Coverslips containing different cell preparations were fixed in 2.5% glutaraldehyde in 0.1 M cacodylate buffer for 2 h at room temperature. For transmission EM, the cells were post-fixed in 2% OsO_4_ (Sigma, St. Louis, MO, USA) in 0.1 M buffer and then counterstained with aqueous 2% uranyl acetate. Dehydration was performed in a graded ethanol series followed by acetone. Monolayers of cells were flat embedded in Epon resin (Sigma, St. Louis, MO, USA) and ultrathin sections were stained with lead citrate. Images were collected using a transmission electron microscope Tecnai G2-12 - SpiritBiotwin FEI - 120 kV.

For scanning EM, coverslips were post-fixed by the OTO (osmium - tanic acid – osmium - Sigma, St. Louis, MO, USA) method, dehydrated in graded ethanol series and dried at critical point under CO_2_. The samples were coated with 3 nm of gold and the images were acquired in the scanning electron microscope (FEG - Quanta 200 FEI).

### DNA and LDH Quantification

DNA was quantified in the supernatants of the cultures, under the different conditions, using the PicoGreen dsDNA kit (Invitrogen, San Jose, CA, USA) according to manufacturer’s instructions.

To quantify LHD in culture supernatants, we used the LDH UV kit (kindly offered by Bioclin, Belo Horizonte, MG, Brazil), according to manufacturer’s instructions.

### Flow Cytometry Analysis of PBMC

Staining was performed as routinely done by us (32). Briefly, wells were harvested, plated at 2 × 10^5^ cells/well, incubated with a 40-μl mix of monoclonal antibodies CD4-Percp-Cy5.5, anti-CD8-APCCy7 or anti-CD69-PE for 15 min at 4°C, washed with PBS, and fixed for 20 min with 2% formaldehyde/PBS. Then cells were washed twice, permeabilized by incubation for 15 min with 0.5% saponin solution, washed, and subjected to intracellular staining with anti-TNF-APC, anti-CD107a-FITC monoclonal antibodies for 20 min at room temperature. Samples were washed twice with 0.5% saponin solution, resuspended in PBS and read on a flow cytometer. To stain with annexin V and propidium iodide (PI), culture cells were washed with PBS and incubated for 15 min with annexin V. After washing, PI was added just prior to acquisition on the flow cytometer. Samples were acquired on the FACS CANTO II and analyzed using FlowJo software (Tree Star, Ashland, OR, USA). All antibodies were from BioLegend (San Diego, CA, USA).

### Time Series for Visualizing CD8-Derived Extracellular Traps and Cell Death

To produce the movies (Time-series), CD8+ T lymphocytes were purified as described above. Non-CD8 cells, leftover from the purification of CD8+ cells, were stained with CFSE as described above, and used as target cells. A proportion of 1:4 CD8+ cells (8 × 10^4^ cells) to 20% non-CD8 cells used as targets (2 × 10^4^ cells) were plated in 24-well plates pretreated with poly-L-lysine and stimulated with anti-CD3/CD28. After 14 h of incubation, ethidium homodimer (EthD-1) (final concentration of 4 µM - LIVE/DEAD ® Viability/Cytotoxicity Kit for mammalian cells - Molecular Probes. Eugene, Oregon, USA) and ionomycin (final concentration of 500 ng/ml; Sigma Aldrich, San Luis, Missouri, USA) were added. A Zeiss 5 Live confocal was used and images were captured every 10 s with CFSE (ex/em ~ 495 nm/~ 515 nm) and EthD-1 (ex/em ~ 532 nm/~ 635 nm) filters. The latter can enter cells with damaged membranes and undergoes a 40-fold increase in fluorescence when binding to nucleic acids, producing a bright fluorescence. The movies were produced using the *Free Microscope Software ZEN blue edition from ZEISS Microscopy* at a rate of 30 frames per second.

### Statistical Analysis

The paired t-test was used to verify differences between Gaussian-distributed data and comparing the same samples under different experimental conditions. Unpaired T test was used to compare amongst different groups. Differences that returned values of p≤0.05 were considered statistically significant. Results were presented as mean+/-standard errors, as samples fell into the normal distribution using Kolmogorov-Smirnov test. All correlation analyses were performed using the Spearman test.

## Results

### Human T Lymphocytes Release Extracellular DNA via Etosis

**Figure 1:**
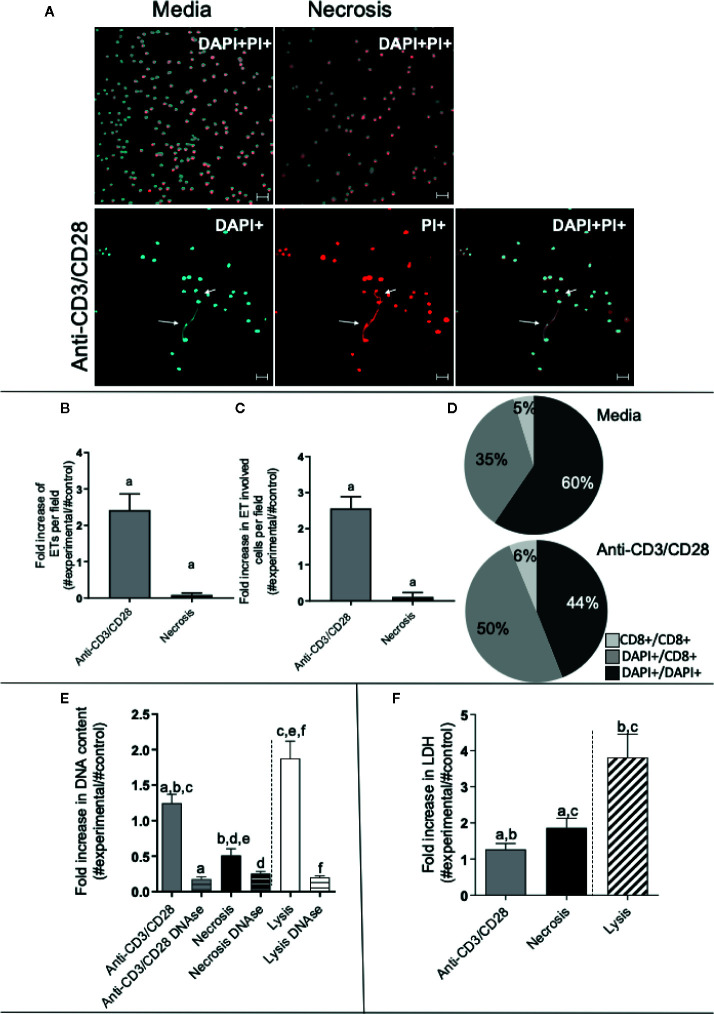
Anti-CD3/anti-CD28 stimulation leads to the release of extracellular traps by T-lymphocyte enriched cultures. **(A)** Representative confocal image of non-stimulated culture (media), necrosis-induced culture (necrosis) and culture stimulated with anti-CD3/anti-CD28 stained with DAPI (light blue) and propidium iodide (PI - red). Arrows highlight extracellular DNA (extracellular traps – ETs) observed in the stimulated cultures. Bars = 20 μm. **(B)** Quantification of ETs after stimulation or induction of necrosis. **(C)** Quantification of the number of cells participating in the ETs in each group. **(D)** Determination of the frequency of ETs connecting DAPI+ cells to DAPI+ cells (DAPI+/DAPI+), any DAPI+ cells to CD8+ cells (DAPI+/CD8+) and CD8+ cells to CD8+ cells (CD8+/CD8+), as indicated, in non-stimulated and anti-CD3/anti-CD28 stimulated cultures. **(E)** Double-stranded DNA was quantified in the culture supernatant using the PicoGreen dsDNA kit (Invitrogen), according to the manufacturer’s instructions. **(F)** Evaluation of necrosis by quantification of LDH release. The release of LDH into the culture supernatant was assessed by spectrophotometry (340 nm) using a commercial kit (Bioclin), according to the manufacturer’s instructions. Data is presented as average per group ± standard error of the mean. Same letters in different bars represents statistically significant differences between them.

We stimulated lymphocyte-enriched PBMC (~72% CD4+ and CD8+ T cells) from healthy individuals with anti-CD3 and anti-CD28 antibodies. This antibody combination provides a T cell specific stimulation that does not directly stimulate other cells in the culture (34, 35). Following 24 h of stimulation, extracellular DNA was detected by staining with DAPI and PI and analyzed by confocal microscopy. **Figure 1A** (top panels) display representative images of unstimulated preparations, as well as preparations in which necrosis was induced by heat, showing that DNA staining (DAPI+ and PI+) was focused inside the cells in both cases. **Figure 1A** (bottom panels) demonstrates that cultures stimulated with anti-CD3/anti-CD28 released extracellular DNA strands associated with cells as seen by co-staining with DAPI and PI (arrows). Quantification of the extracellular DNA strands (extracellular traps – ETs) showed that ETs appear more frequently in anti-CD3/anti-CD28-stimulated cultures, as compared to cultures in which necrosis was induced (p < 0.05) (**Figure 1B**). In addition, the number of cells involved in ETs formation was also higher in anti-CD3/anti-CD28-stimulated cultures (**Figure 1C**), as compared to necrotic preparations (p < 0.05). Interestingly, stimulation with anti-CD3/anti-CD28 increases the number of CD8+ cells associated with formation of ETs (the sum of %CD8/DAPI plus %CD8/CD8 increases from 40.5% to 56%) (**Figure 1D**).

To quantify the amount of DNA released by lymphocyte-enriched preparations stimulated with anti-CD3/anti-CD28, we measured the DNA in culture supernatants using the PicoGreen dsDNA kit, as described in Materials and Methods. While all preparations released DNA in the supernatant, the release by anti-CD3/anti-CD28-stimulated cells was two-fold higher than that seen from the necrosis group (**Figure 1E**). Lysed cells (positive control) had the highest DNA titers (**Figure 1E**). The DNA signal was abolished by treatment with DNAse in all preparations, as expected (**Figure 1E**).

We measured the release of lactate dehydrogenase (LDH), an important molecule classically associated with necrosis (36). The necrosis cultures presented greater release of LDH than the anti-CD3/anti-CD28-stimulated cultures (**Figure 1F**). Cell lysis was used as a positive control, showing high release of LDH. Thus, DNA release was higher in anti-CD3/anti-CD28 stimulated cultures as compared to necrosis, whereas LDH release was higher in the group where necrosis was induced, showing an important difference between the two processes.

We also evaluated the occurrence of apoptosis and total cell death in the cultures by staining with Annexin V and/or propidium iodide (PI) using flow cytometry. The frequency of total lymphocyte death was determined within the lymphocyte gate and corresponded to the sum of total PI+ cells and total Annexin V+ cells. Non-stimulated and anti-CD3/anti-CD28-stimulated cultures had a low occurrence of cell death as compared to necrosis and staurosporin-treated cultures (**Supplementary Figure 1A**). Occurrence of apoptosis, as measured by the frequency of Annexin V+ PI- cells, was low in all cultures (except for the staurosporin control), although also higher in the necrosis group as compared to media and anti-CD3/CD28 (**Supplementary Figure 1B**). Occurrence of necrosis (as measured by AnnexinV^low^ PI+ cells) was low in all cultures, except for the necrosis-induced cultures (**Supplementary Figure 1C**).

### Electron Microscopy Shows Ultrastructural Characteristics Suggestive of Etosis in Anti-CD3/Anti-CD28 Stimulated Cells

**Figure 2:**
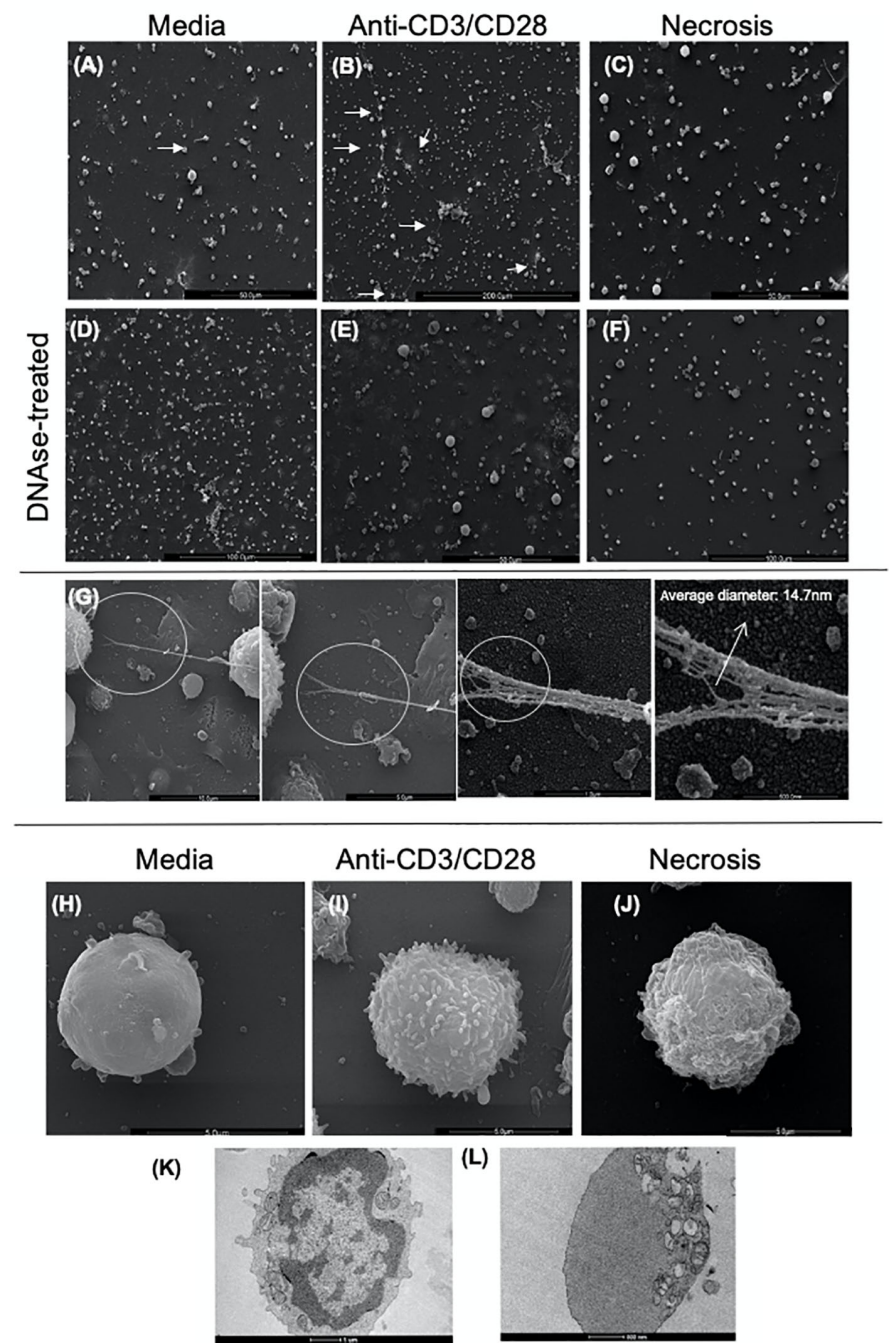
Scanning and transmission electron microscopy of cultures enriched in T lymphocytes show the presence of extracellular traps (ETs). Representative scanning electron microscopy images of **(A)** Non-stimulated culture (media), **(B)** Culture stimulated with anti-CD3/anti-CD28, **(C)** Necrosis-induced culture (necrosis). In the **(A–C)** images, the arrows indicate structures compatible with extracellular traps (arrows). **(D)** Non-stimulated culture treated with DNAse (DNAse-treated media), **(E)** Culture stimulated with anti-CD3/anti-CD28 treated with DNAse, **(F)** Culture of cells induced to necrosis treated with DNAse (DNAse-treated necrosis). **(G)** Detail of an ET (extracellular DNA) present in a culture enriched with T lymphocytes after anti-CD3/anti-CD28 stimulation. High-resolution SEM analysis of ETs that consist of fibers with an average diameter of 14.7 nm, arrow. Scanning electron microscopy images of cell topography under different conditions, showing the distinction amongst them: media **(H)**, anti-CD3/anti-CD28 **(I)**, and necrotic **(J)**. Transmission electron microscopy of **(K)** unstimulated T cell; **(L)** cell in etosis present in culture stimulated with anti-CD3/anti-CD28.

We performed scanning (SEM) and transmission electron microscopy (TEM) to visualize the DNA-composed structures and cell morphology in lymphocyte-enriched cultures stimulated with anti-CD3/anti-CD28 or induced to necrosis. **Figures 2A–C** show the presence of fine structures that seem to connect one cell to another, as indicated by arrows, observed in SEM. The observed structures were clearly more evident in anti-CD3/anti-CD28-stimulated cultures (**Figure 2B**). **Figures 2D–F** show that treatment with DNAse abolished the presence of such structures. **Figure 2G** shows the detail of a DNA-formed structure derived from an anti-CD3/anti-CD28-stimulated culture. The average thickness of each filament that formed the structures was 14.7 nm, consistent with a DNA filament. **Figures 2H–J** show the morphology of typical lymphocytes observed in non-stimulated cultures, anti-CD3/anti-CD28-stimulated cultures and necrosis induced cultures, respectively.

TEM images of unstimulated cells showed aspects of typical resting lymphocytes with an intact nuclear envelope and organelles homogeneously dispersed in the cytoplasm (**Figure 2K**). In contrast, cells from anti-CD3/anti-CD28-stimulated cultures displayed a dramatically different morphology consistent with etosis (11), showing a lack of nuclear envelope and accumulation of organelles on one side of the cell (**Figure 2L**). These data clearly show the presence of DNA filament structures released by stimulated T cells with ultra-structural aspects compatible with the occurrence of etosis.

### Extracellular DNA-Containing Structures Co-Stain With Anti-Histone Monoclonal Antibodies, but Not With Phalloidin, an Actin Marker

**Figure 3:**
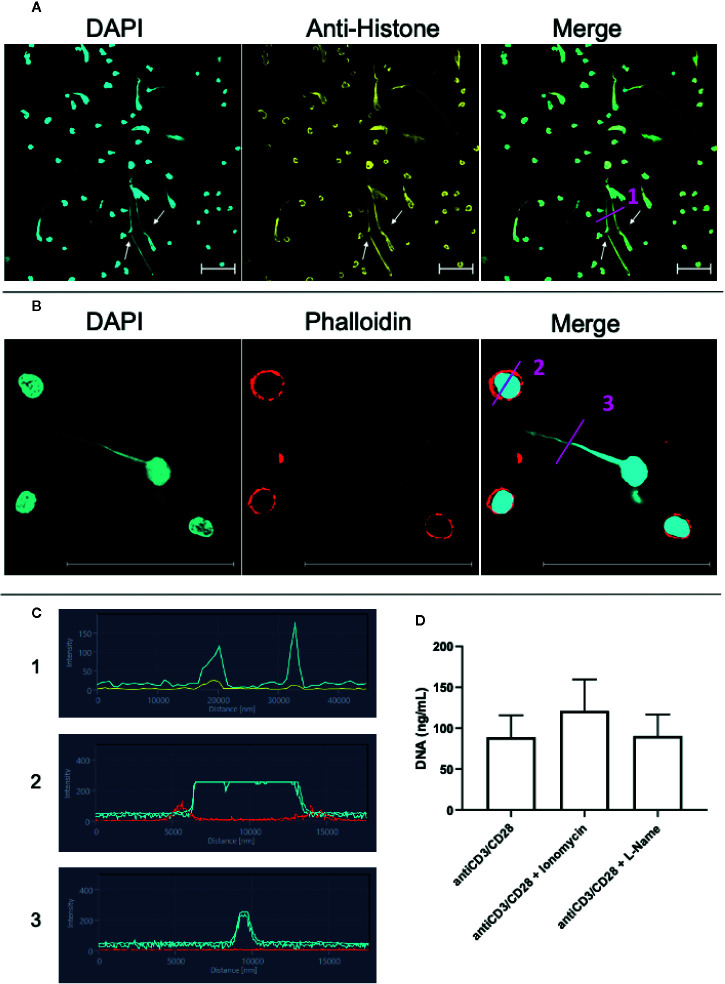
Lymphocytes are able to release extracellular traps (ETs) composed of DNA and histones. T lymphocyte-enriched preparations were plated on poly-L-lysine coverslips, stimulated or not with anti-CD3/anti-CD28 and maintained in culture for 24 h. The preparations were stained with DAPI (light blue) and/or anti-histone (yellow), and/or phalloidin (red - actin evidence). Bars = 50 µm. **(A)** Representative confocal image of culture stimulated with anti-CD3/anti-CD28 and labeled with DAPI (light blue)/anti-histone (yellow). Arrows highlighted extracellular traps observed in the stimulated cultures. **(B)** Samples were stained with DAPI (light blue)/phalloidin (red). **(C)** shows histograms derived from the details marked with (1), (2) and (3) in panels **(A, B)**, showing the co-localization of DAPI and histones (1) and the lack of co-localization of DAPI and actin in the cell body (2) and in the extracellular DNA-structure (3). **(D)** shows the effect of ionomycin and L-NAME in the release of extracellular DNA by anti-CD3/anti-CD28 stimulated cultures.

The presence of histones is a hallmark of ETs (10, 11). Thus, we performed a staining combining DAPI and anti-histone monoclonal antibodies. These two markers clearly co-localized on the extracellular structures in anti-CD3/anti-CD28 stimulated cultures, demonstrating the presence of ETs (**Figure 3A** in immunocytochemistry, and **Figure 3C**-in fluorescence histogram 1). The histogram shows that DAPI (blue line) and histone (yellow line) co-localize.

In order to exclude the possibility that the long DNA-containing structures were tunneling nanotubes or cytonemes, structures involved in cell communication that contains DNA and actin (37–39), we co-stained the samples with phalloidin, an actin-detecting molecule, and DAPI. **Figure 3B** clearly shows that the extracellular DAPI+ DNA structures do not co-stain for phalloidin, thereby excluding the presence of actin in the structures, and ruling out the possibility that they are tunneling nanotubes or cytonemes. This result is also observed in histograms 2 and 3 in **Figure 3C**. The histograms show that phalloidin (red) and DAPI (blue) staining do not overlap.

Arginine deaminases 4 (PAD4) is fundamental for the occurrence of etosis in neutrophils and is responsible for DNA unfolding (40, 41). PAD4 activity is increased by intracellular Ca^++^, which is elevated by the calcium ionophore ionomycin (42). iNOS activity has also been correlated with ET release by neutrophils (40). **Figure 3D** shows that ionomycin treatment in lymphocyte-enriched cultures increases DNA release by 36% (p= 0.07, as compared to anti-CD3/CD28 stimulated cultures), suggesting that Ca^++^ is also associated to DNA release by lymphocytes. On the other hand, blocking of iNOS activity by L-N^G^-Nitro arginine methyl ester (L-NAME) does not alter the release of ETs in lymphocyte-enriched cultures (**Figure 3D**).

### Human CD8+ T Cells Release Extracellular Traps That Are Morphologically Distinct From the Ones Released by CD4+ T Cells

**Figure 4:**
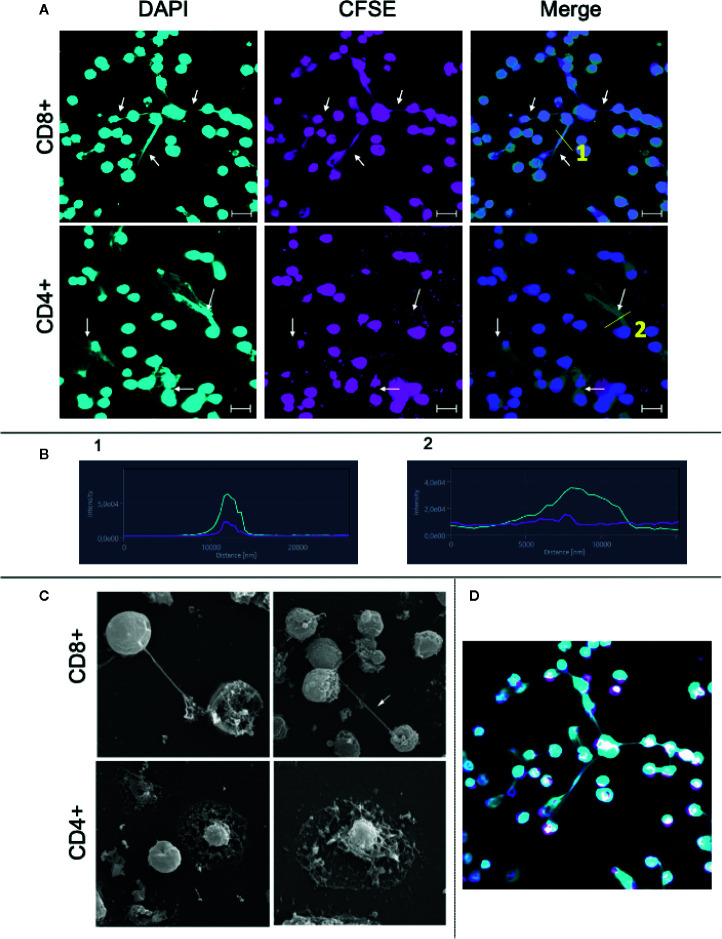
Purified CD8+ and CD4+ T lymphocytes release extracellular traps (ETs) upon stimulation. Purification of CD8+ and CD4+ T lymphocytes was done by sorting as described in Material and Methods. **(A)** CD8+ and CD4+ lymphocytes were stained with CFSE, plated on poly-L-lysine coverslips and stimulated with anti-CD3/anti-CD28 for 24 h. After incubation the cells were stained with DAPI as described in the Materials and Methods. Representative images from confocal microscopy analyses for show the DAPI+ (light blue) cells, CFSE+ (pink) cells and for both. Arrows highlight the extracellular traps. Bars = 20 μm. **(B)** shows histograms derived from the details marked with (1) and (2) in panels **(A)**, demonstrating the co-localization of DAPI and CFSE in CD8-derived and CD4-derived ETs, respectively. **(C)** CD8+ and CD4+ cells were stimulated and processed for SEM analysis, as described. Lymphocytes cells were plated on poly-L-lysine coverslips, stimulated with anti-CD3/anti-CD28 and maintained in culture for 48 h. The left CD8+ and CD4+ images have a magnification of 3,000× while the right ones, 5,000×. Arrow: structure connecting cells. **(D)** Confocal image obtained by staining with CFSE (pink) and DAPI (blue, as above) showing CD8+ T cell-derived DNA filament connecting cells.

We then focused on studying whether CD8+ T cells can release ETs. CD8+ T cells were purified using a cell sorter, stimulated with anti-CD3/anti-CD28, and analyzed using confocal and electron microscopy. As a comparison, CD4+ cells were also purified and submitted to the same culture conditions. **Figure 4A** (top panels) show that stimulated CD8+ T cells release ETs as filaments connecting neighboring cells (arrows). In contrast, CD4+ T cells release ETs with a diffuse pattern around the cells (**Figure 4A**, bottom panels, arrows). **Figure 4B** shows that both CD8+ and CD4+ derived ETs are composed mainly of DNA (DAPI+, blue intensity curve), but also contain some cytoplasm proteins (CFSE+, pink intensity curve). Scanning microscopy analysis confirmed that activated CD8+ T cells released long and thin structures, often connecting one cell to another (**Figure 4C**, arrow in top panels, **Figure 4D**, and **Supplementary Video 1**). In contrast, CD4+ T cells release DNA around the cell, forming a halo (**Figure 4C**, bottom panels). CD4+ T cells appeared to have a disintegrated membrane, suggesting cell death.

To investigate the activation state of the stimulated cells, as well as the occurrence of apoptosis in the different cultures, we evaluated the expression of CD69 and TNF as indicators of activation, and AnnexinV/PI as indicators of apoptosis.

**Figure 5:**
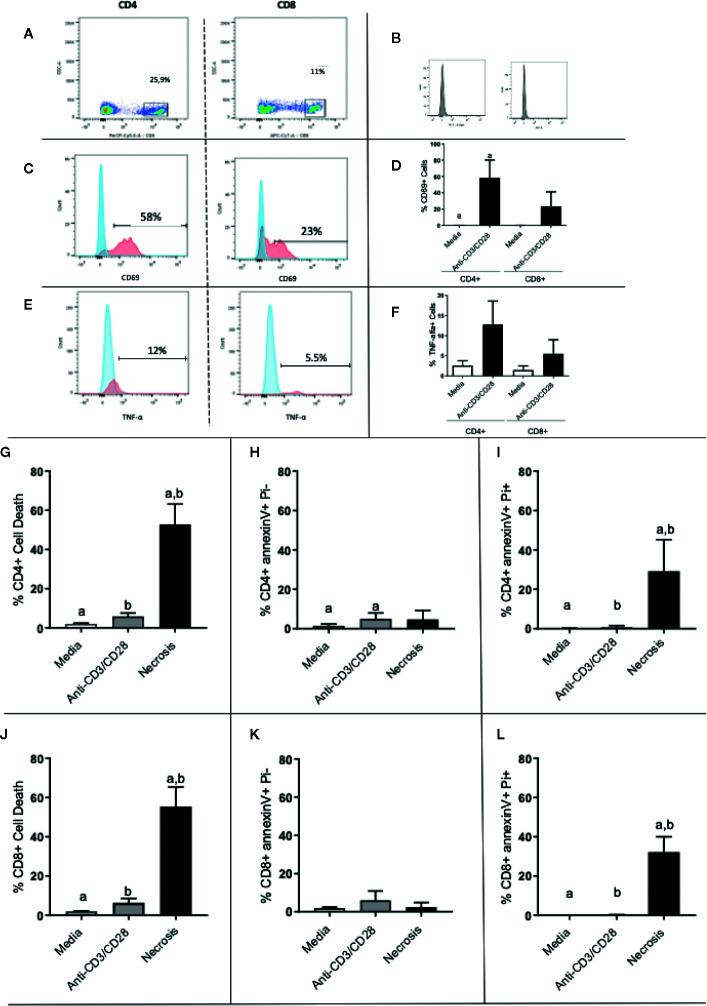
Evaluation of activation and death of CD4+ and CD8+ T cells. **(A)** Gate selection for CD4+ and CD8+ analysis; **(B)** isotype controls; **(C, E)** representative histograms for CD69 and TNF analysis, respectively, in CD4 and CD8 cells, as indicated. Expression of CD69 and **(D)** TNF **(F)** by CD4+ and CD8+ T lymphocytes before and after stimulation with anti-CD3/anti-CD28. **(G–I)** show total death, early apoptosis and late apoptosis of CD4+ T cells, respectively. **(J, L)** show total death, early apoptosis and late apoptosis of CD8+ T cells, respectively. Data is presented as average per group ± standard error of the mean and presence of the same letters in different bars represents statistically significant differences between them (p<0.05).

**Figure 5A** shows the selection of lymphocyte subpopulations to be analyzed in scatter plots (granularity x CD4+ or granularity x CD8+) obtained by flow cytometry. **Figure 5B** shows the isotype controls. **Figures 5C**, **E** show representative histograms of CD69 and TNF-alpha, respectively. After 24 h of stimulation, we observed that both CD4+ and CD8+ T cells had a higher expression of CD69 when compared to media control (**Figure 5D**). Interestingly, activated CD4+ T cell cultures exhibit a much greater frequency of TNF-alpha+ cells as compared to non-stimulated cultures, than CD8+ T cell cultures (**Figure 5F**). The overall frequency of cell death for CD4+ and CD8+ T cells (**Figures 5G, J**, respectively), as well as necrosis (**Figures 5I, L**, respectively), was low in stimulated cultures. While the frequency of cells in apoptosis was also low, it was higher in CD4+ T cell stimulated cultures, as compared to non-stimulated ones (**Figure 5H**). This statistically significant difference was not observed in cultures of CD8+ T cells (**Figure 5K**). This suggests that the higher production of TNF observed in CD4+ T cells may be associated with the higher apoptosis observed in CD4+ T cells, as this cytokine may induce apoptosis.

### Release of Extracellular DNA by CD8+ T Cells Is Followed by Death of Target Cells

**Figure 6:**
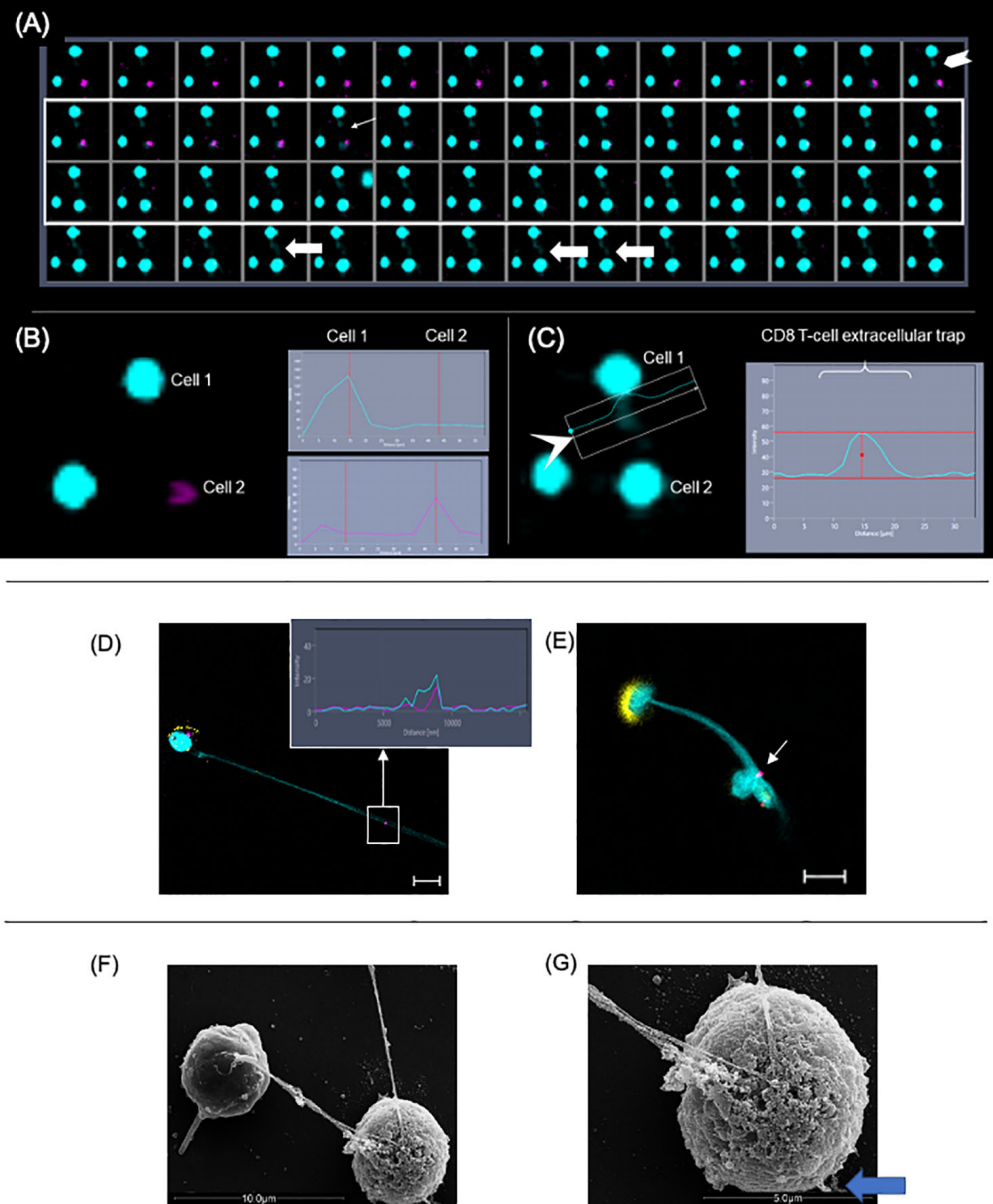
Extracellular DNA from CD8+ T cells induces death of neighboring cells. Purified CD8+ T cells were cultured with CFSE-labeled targets (pink) at a ratio of 1:4. Cultures were stimulated with anti-CD3/anti-CD28+ionomycin and stained with live-dead marker (EthD-1). Images were obtained in 10-s intervals using excitation/emission capture of 495/515 nm for CFSE and 532/635 for EthD-1, on a Zeiss 5-live microscope. **(A)** Static images showing the moment in which the liberation of extracellular DNA by a CD8+ T cell (chevron, last frame in first row) is followed by the conversion of a pink cell (target) into a blue cell (light arrow at frame 5, second row), indicating death. **(B)** Fluorescence histograms showing cell 1 (EthD-1- blue) and cell 2 (CFSE-pink) before the release of extracellular DNA by cell 1. **(C)** Fluorescence histogram showing extracellular DNA from cell 1. In this detail, it is possible to see the DNA strand extruding toward cell 2. This can also be observed in **(A)** in frames 4, 8 and 9 from last row (fat arrows). **(D, E)** Representative immunofluorescence image of T-lymphocyte enriched cultures stained with anti-CD8 (yellow), anti-CD107a (pink), DAPI (light blue). Arrow indicates that CD107a+ vesicles co-localized with the extracellular trap (DAPI+). Bars = 10 μm. Detail shows histogram demonstrating the co-localization of CD107a+ and DAPI+ staining. **(F)** Scanning electron microscopy image showing two cells connected by a DNA filament. **(G)** The SEM detail shows the telltale morphology of an apoptotic cell with a ruffled and blebbed membrane (blue arrow).

To confirm the dynamics of LET release by CD8+ T cells in real-time, we performed time-frame videos (**Figure 6A** as a static figure and **Supplementary Video 2**). CD8+ T cells were purified and cultured with CFSE-stained target cells at a ratio of 1:4, as described in *Methods*. **Figure 6A** shows a CD8+ T cell-derived extracellular DNA filament forming (chevron, last frame of first row), toward a CFSE-labeled live cell (pink), and its subsequent death (thin arrow fifth frame in second row from top shows the cell transitioning from pink to blue; the following frame shows the cell in blue). Arrows in last row show the extracellular DNA connecting both cells. **Figure 6B** shows the histogram for the fluorescence of ethidium homodimer (EthD-1) obtained from image in first row, which is positive in cell 1 (CD8+ T cell, blue) indicating loss of membrane integrity, and negative in cell 2 (target cell, still alive), which is stained with CFSE (pink). **Figure 6C** shows the histogram for the extracellular DNA released from cell 1, and also shows that cell 2 is now positive for EthD-1, depicting its loss of membrane integrity after contact with the CD8+ T cell-derived LET. This sequence can be visualized in **Supplementary Video 2** and shows that the release of extracellular DNA by CD8+ T cells is followed by the death of the neighboring cell.

### CD8+ T Cell-Derived LETs Carry CD107a+ Vesicles

Next, we sought to determine if the LETs could carry cytotoxic signals by examining the physical association of cytolytic vesicles expressing CD107a with CD8+ T cell-derived LETs. We observed that the expression of CD107a (**Figure 6D**, pink, in square) colocalized with DAPI-stained LETs (**Figure 6D**, blue, in square) coming from CD8+ T cells (**Figure 6D**, yellow), and being delivered to a distant cell (**Figure 6E**, arrow). Interestingly, the CD8+ T cell releasing the LET also exhibits a dramatic polarization of the CD8 molecule on the opposite pole from where the LET is released (**Figure 6E**). Co-localization of CD107a and LET is also seen in the histogram insert relative to the section highlighted by the square in **Figure 6D**. Ultrastructural analysis shows that one of the cells connected to the extracellular DNA displays the telltale topology of an apoptotic cell (**Figures 6F, G**), with membrane blebs (blue arrow in **Figure 6G**). Together, these data suggest that the delivery of CD107a+ vesicles by the launched CD8+ T cell-derived LETs may be involved in the observed cell death.

### LETs Are Associated With Increased Tissue Pathology in Human Leishmaniasis

After demonstrating that CD8+ T cells can release LETs, we sought to investigate if this phenomenon was associated with the pathology of human tegumentary leishmaniasis, a disease in which CD8+ T cells play a key role in pathology. Thus, we focused our study on LETs derived from CD8+ T cells in cutaneous and mucosal forms of leishmaniasis.

**Figure 7:**
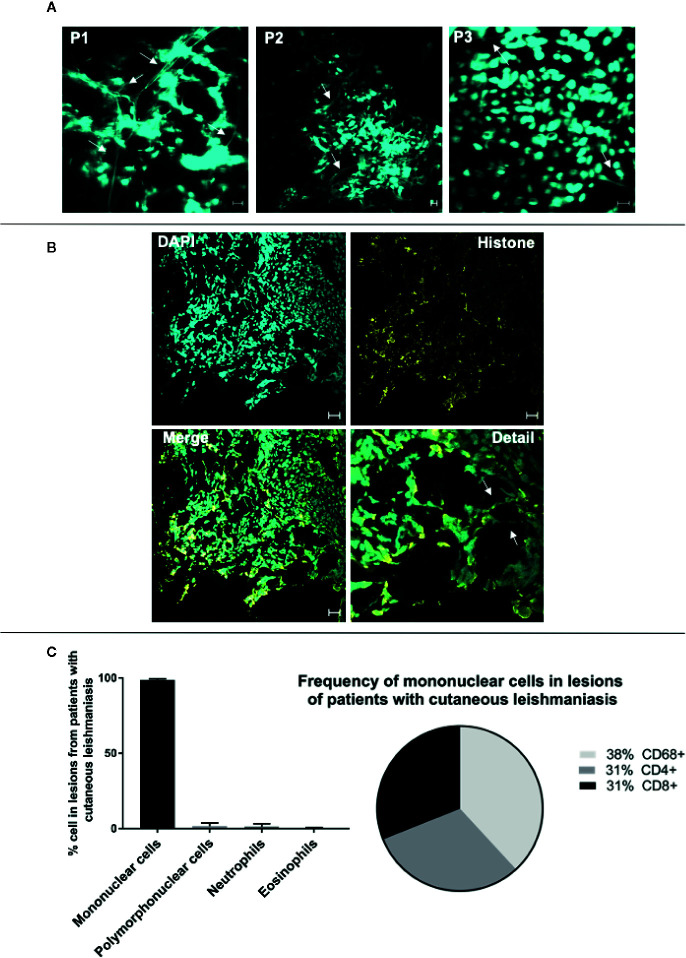
Lesions of patients with cutaneous leishmaniasis display extracellular DNA. Tissue sections were stained with DAPI or Alexa 488-labeled anti-histone monoclonal antibodies as described in *Materials and Methods*. **(A)** Representative confocal images of lesions from patients with cutaneous leishmaniasis stained with DAPI alone (P1, P2 and P3 represent three different patients), showing the occurrence of extracellular DNA structures. The bars = 10 μm. **(B)** Representative lesion from a cutaneous leishmaniasis patient stained with DAPI and anti-histone monoclonal antibody. In detail, colocalization of the DAPI (light blue) and histone (yellow). Arrows show extracellular DNA co-stained with DAPI and anti-histone monoclonal antibody. Bars = 20 μm. **(C)** Evaluation of cellular composition of lesions from cutaneous leishmaniasis patients (n=14) through histopathology analysis using hematoxylin/eosin stain (graph), and confocal microscopy using anti-CD4, anti-CD8 and anti-CD68 monoclonal antibodies (pie chart), as described in *Materials and Methods*.

Previous studies have shown that skin lesions from individuals infected with Leishmania displayed neutrophil-derived ETs (14, 22). In addition, we have demonstrated that CD8+ T cells play a key role in the development of cutaneous leishmaniasis (CL) lesions (24, 26, 27), and transcriptome analysis from patient lesions has supported the existence of an inflammatory CD8+ cytolytic T cell response in lesion microenvironment (43, 44). While further investigating cellular mechanisms of tissue destruction in leishmaniasis, we observed, using confocal microscopy, that lesions from CL patients infected with *L. braziliensis* were dense with what appeared to be DNA filaments as evidenced by DAPI staining (**Figure 7A**, arrows). We further stained the lesions with DAPI and anti-histone antibodies concomitantly, and observed the co-localization of extracellular DNA and histones, suggesting that the observed structures were indeed ETs (**Figure 7B**, overall and arrows). Importantly, we also determined that the inflammatory infiltrate of those lesions was predominantly composed of mononuclear cells, with a very low frequency of polymorphonuclear cells (**Figure 7C**), and that over 60% of the inflammatory infiltrate was composed of CD4+ and CD8+ cells (**Figure 7C**). Thus, the presence of DNA+histone+ structures outside the cells, our in vitro findings demonstrating CD8+ T cell produced LETs, together with T cell dominant lesions from *L. braziliensis*-infected individuals in the virtual absence of neutrophils (our own study, as well as a lack of transcriptome profiles consistent with neutrophils – *44*), led us to investigate whether T cell released LETs in cutaneous leishmaniasis lesions, and if they were related to disease progression and severity.

The intensity of the inflammatory infiltrate present in lesions from patients with CL and ML is a measure of pathology severity in these diseases (24–26). As expected, our analysis of this cohort confirmed that disease progression from early to late in CL and the more severe ML form displays a more intense inflammatory infiltrate (**Supplementary Figure 2A**). We show here that the number of CD8+ cells (**Supplementary Figure 2B**), as well as the number of CD8+CD107+ cells (**Supplementary Figure 2C**) display a positive correlation with the intensity of the inflammatory infiltrate in the evaluated TL disease forms.

**Figure 8:**
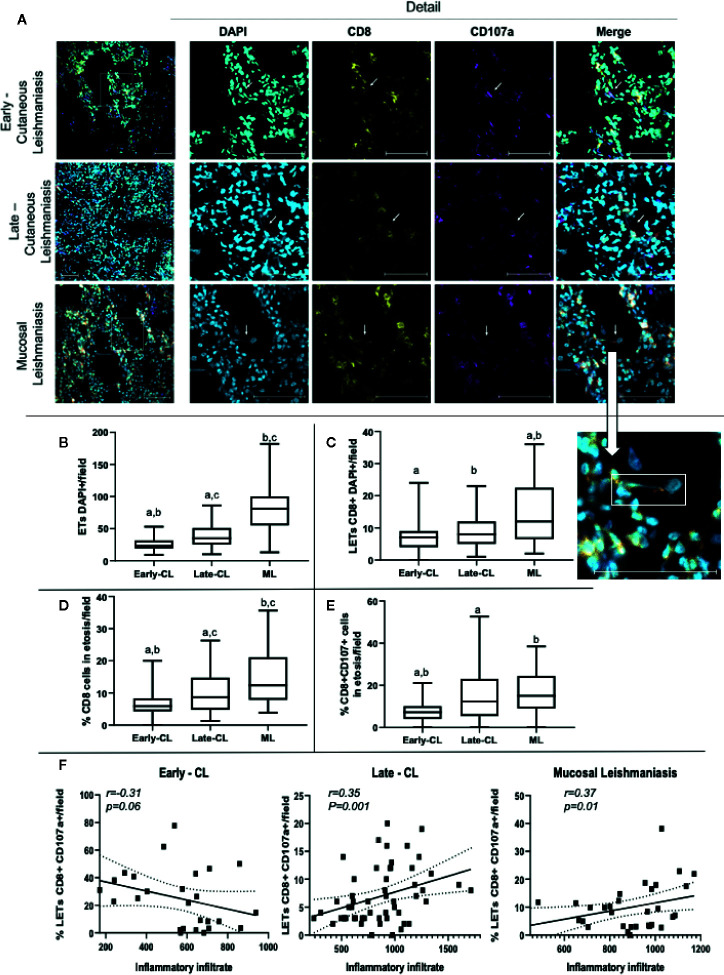
Lesions of patients with early and late cutaneous, as well as mucosal leishmaniasis, display extracellular DNA, CD8+ cells and CD107a expression. Tissue sections were stained with DAPI, anti-CD8-PE and anti-CD107a-FITC monoclonal antibodies as described in Materials and Methods. **(A)** Representative confocal images of lesions from patients with early (n=2; 27 fields) and late cutaneous leishmaniasis (n=4; 43 fields), and mucosal leishmaniasis (n=2; 28 fields) stained with DAPI alone, anti-CD8 alone, anti-CD107 alone and a merged image of the three stainings (right panels). The detail image shows extracellular DNA co-localized with CD107a+ vesicle, connected to a cell. The bars = 50 μm. **(B)** number of total ETs; **(C)** number of CD8+ cells connected to LETs; **(D)** percentage of CD8+ cells in etosis within the total CD8+ population; **(E)** percentage of CD8+CD107a+ cells in etosis within the total CD8+CD107a+ sub-population. Data is presented as average per group ± standard error of the mean and presence of the same letters in different bars represents statistically significant differences between them (p<0.05). **(F)** Correlation analysis between the number of inflammatory cells/lesion field and the frequency of CD8+CD107+LETs/field for each clinical form.

In order to investigate the association between the presence of LETs with the progression and severity of tegumentary leishmaniasis (TL), we compared the number of DNA strands in lesions from patients with early versus late CL lesions, and of CL versus mucosal (ML) lesions. We also evaluated the participation of CD8+ T cells, as well as CD8+CD107a+ cells in the release of LETs in these lesions. **Figure 8A** shows representative staining with DAPI, CD8 and CD107a in lesions from patients with different stages of CL or with ML, clearly showing the existence of CD8-associated LETs, colocalizing DNA strands and CD107a. We observed that progression from early to late CL lesions was accompanied by an increase in the number of overall ETs, and that the same was observed when comparing both stages of CL versus the more severe form, ML (**Figure 8B**). Examining the number of CD8 cells involved in the release of LETs, we observed similar numbers comparing early and late CL lesions, but greater numbers when comparing more severe ML to CL lesions (**Figure 8C**). The percentage of CD8 cells undergoing etosis was related to the progression from early to late, as well as when comparing CL to the more severe ML (**Figure 8D**). A significant increase in the percentage of CD8+CD107a+ cells in etosis was observed with lesion progression and severity (**Figure 8E**).

In order to determine if LETs are also associated with the in situ inflammatory pathology in the different clinical forms, we performed a correlation between the number of cells present in the inflammatory infiltrate, as a measure of the intensity of the inflammation, versus the frequency of CD8+CD107+ cells releasing LETs. Interestingly, we observed that while the frequency of CD8+CD107+ cells in etosis was not correlated with the inflammatory infiltrate in early stages of CL, the frequency of CD8+CD107+ cells in etosis was correlated with the intensity of the inflammatory infiltrate as CL progressed (late CL), and also with the severe ML form (**Figure 8F**), clearly associating release of LETs by CD8+CD107+ cells with disease progression and severity.

## Discussion

Our study demonstrates for the first time that human CD8+ T lymphocytes release extracellular traps (LETs) upon stimulation, and that these LETs are morphologically distinct from the ones released by CD4+ T cells. Importantly, ETs derived from CD8+ T cells form long DNA filaments connecting one cell to another, contain CD107a+ vesicles, and induce death in cells that enter in contact with it. This suggests that release of ETs by CD8+ T lymphocytes may represent a mechanism of delivering cytotoxic vesicles to distant targets.

Brinkmann et al. (10) first demonstrated that ETs were composed mainly of DNA and histones. Other authors also characterized ETs produced by different cells as containing mainly DNA (13, 15, 45). Here we demonstrated that the structures released by anti-CD3/anti-CD28 stimulated lymphocytes are also composed principally of DNA since: (1) they have a structure and diameter similar to a DNA filament as shown by SEM, (2) treatment with DNAse eliminates the structures observed in SEM, (3) they stain with DAPI, PI and anti-histone antibodies, and (4) supernatants from stimulated cultures contain DNA. Together, these data allow us to conclude that lymphocyte-derived ETs (LETs) are also composed of DNA as described for ETs derived from neutrophils and other cell types. While previous work has suggested that lymphocytes can release extracellular DNA (16), our work brings a more in-depth characterization of these structures.

Given necrosis can also lead to DNA release (46, 47), it was critical to demonstrate that the observed LETs were not related to necrosis. To test this possibility, we first demonstrated that cell death, particularly necrosis, was extremely low in the stimulated cultures. In addition, the release of the enzyme lactate dehydrogenase (LDH), which is characteristic of necrosis (36), was significantly lower in the supernatant of anti-CD3/anti-CD28-stimulated cultures as compared to the necrosis-induced, and positive control groups (lysed cells). We also observed that anti-CD3/anti-CD28-stimulated cultures led to a greater DNA release than the cultures induced to necrosis. Moreover, DNAse I treatment decreased DNA quantification in the supernatants from anti-CD3/anti-CD28-stimulated cultures, confirming that the DNA collected in the culture supernatant was double-stranded DNA, as previously shown in other models of etosis (14, 48). Necrosis activates intracellular DNAse to cleave DNA, which explains the low quantification of DNA released in necrosis (49). The presence of LETs was detected using DAPI and PI, which is a DNA intercalating agent, used in other studies to show the presence of DNA in ETs (46). In addition, co-localization of extracellular DNA and histones by immunofluorescence (10, 13) in cells from the stimulated cultures confirmed the occurrence of LETs, formed by nuclear DNA.

Previous studies have demonstrated that Ca^++^ mobilization and production of reactive oxygen species (ROS) are associated with the release of ETs by neutrophils (21, 41, 50). Here we showed that the release of LETs is associated with the increase of Ca^++^, as the use of a Ca^++^ ionophore (ionomycin) increased the release of LETs. On the other hand, inhibition of nitric oxide synthase, important in the generation of ROS, did not alter the release of LETs. It is possible that NOS activation is related to the release of ETs by innate cells, but not particularly important in lymphocytes, as the expression of this enzyme is greater in innate cells. These analyses demonstrate that the mechanisms involved in the release of LETs share some common and distinct pathways regarding the release of ETs by other cell types.

Quantitative analysis of extracellular DNA demonstrated that anti-CD3/anti-CD28 stimulation induced a greater release of LETs and involved a greater number of cells releasing LETs, as compared to the necrosis group. While anti-CD3/anti-CD28 stimulates all T cells, not all cells in the cultures release ETs. The characteristics of ET-releasing T cells remain to be determined. It is possible that these structures are only released by pre-activated T cells, or by a subset of memory or effector cells. Identification of such sub-populations are beyond the scope of this paper but studies to further characterize ET-releasing T cells are underway in our lab. Given the use of anti-CD3-anti-CD28 does not directly involve MHC-peptide/TCR interaction, we were unable to determine here if antigen-specific interaction triggers the release of LETs. Further studies using antigen-specific responses are necessary to clarify the role of class I-mediated antigen presentation in the process of LET formation.

Cultures stimulated with anti-CD3/anti-CD28 present a greater number of extracellular strands connecting one cell to another, as observed by scanning electron microscopy (SEM). Importantly, DNAse treatment eliminated the strands at the ultrastructural level, thereby confirming their DNA composition. SEM showed morphology consistent with resting, activated and necrotic cells from non-stimulated, anti-CD3/anti-CD28 stimulated and necrosis cultures, respectively. This shows clear differences amongst stimulated and necrosis cultures: the necrosis group had cells with a degraded cell membrane, typical of necrotic cells (51), whereas the activated cells had a ruffled membrane typical of healthy activated cells (51, 52). Also, using SEM, the thickness of the filaments was determined to be 14.7 nm in diameter, compatible with DNA strands (10, 40). Internal morphology analysis, as evaluated by transmission electron microscopy (TEM), showed that resting cells displayed intact plasma and nuclear membranes, with typical heterochromatin, and organelles homogeneously dispersed in the cytoplasm, whereas cells from the activated cultures, in which LETs were observed, displayed morphology similar to that observed in cells in etosis (11), without a visible nuclear membrane and organelles polarized towards one side of the cell.

Tunneling nanotubes (TNTs) and cytonemes, are actin-based structures described as involved in cell-cell communication (37–39). Analysis of actin detection clearly showed that there was no co-localization of actin and DNA staining, excluding the possibility that the structures we observed were TNTs or cytonemes.

Confocal microscopy analysis of cultures using purified CD8+ T cells showed that they released DAPI+ LETs upon stimulation with anti-CD3/anti-CD28. LETs are mainly composed of DNA, but also contain cytoplasmic proteins, as evidenced by CFSE labeling, and as described for ETs from neutrophils and monocytes (10, 15, 53). Interestingly, SEM analysis of the stimulated purified cells showed that the morphology of CD8-derived LETs was dramatically distinct from the ones derived from CD4+ T cells. While CD8-derived LETs were composed of DNA filaments connecting two distant cells, CD4-derived LETs appeared as a “cloud” around the cells. In addition, the CD4+ T cells appeared to have a disintegrated membrane, suggesting cell death. While the overall cell death in cultures was low, the frequency of CD4+ T cells undergoing apoptosis in the stimulated cultures was higher than in non-stimulated ones. In addition, stimulated CD4 cultures produced high levels of TNF, which has been associated with cell death (54). Interestingly, recent work demonstrated that NETs can carry miRNA associated with the regulation of TNF function (55).

CD8+ T cell effector function is associated with cytolytic activity, which involves the delivery of granules containing cytotoxic molecules to the target cells (1). Cytotoxic granules contain cytolytic enzymes, and classically express lysosomal markers, such as LAMP-1 (CD107a) (1, 3, 56). The mechanisms of delivery of cytotoxic granules described to date demonstrate that this process involves cell-to-cell interaction (57). However, the morphological appearance of the CD8-derived LETs connecting one cell to another led us to hypothesize that these LETs could be involved in the delivery of cytotoxic granules to distant cells. To test this, we evaluated the expression of CD107a in cultures of CD8+ T cells stimulated with anti-CD3/anti-CD28, using confocal microscopy. Our data showed a clear co-localization of CD107a with the DAPI+ LETs released by CD8+ T cells. In addition, SEM also showed cells connected to CD8 LETs displaying morphology consistent with that of dying cells. This mechanism of death delivery has implications for CD8 mediated immunoregulation and immunopathology in highly activated inflammatory microenvironments. Not only could CD8+ T cells deliver death to a distant cell, but the release of LETs could trigger a stronger inflammatory cascade, similar to that seen by neutrophil released ETs (14, 58).

After demonstrating in vitro that CD8+ T cells can release LETs, we moved our approach to test if this phenomenon was associated with pathology in a human disease setting. ETs derived primarily from neutrophils have been associated with the pathogenesis of several autoimmune diseases, as well as cancer and diabetes-associated vasculopathy (59–61). In addition, NETs have been associated with leishmaniasis (14, 22). Thus, In order to also verify the relevance of CD8-derived LETs in a disease setting, we studies the occurrence of these structures in human tegumentary leishmaniasis (TL), given previous studies had shown that CD8+ T cell cytotoxicity was associated with tissue pathology in leishmaniasis caused by *L. braziliensis* infection (24–28). Thus, we evaluated if the progression and severity of lesions caused by *L. braziliensis* were associated with occurrence of CD8+ T cell derived LETs. We first performed a double staining using DAPI and anti-histone monoclonal antibodies in lesions from TL patients and observed that DAPI+ and anti-histone+ structures co-localized inside cells (nucleus), as well as outside in strands, suggesting the presence of ETs in lesions from patients infected with *L. braziliensis*. Histopathological analysis of the lesions did not show extensive necrosis as found by others (62), suggesting that extracellular DNA was not derived from necrosis. While previous study showed the presence of NETs in leishmaniasis lesions from individuals from Rio de Janeiro state, other studies had shown that lesions from patients infected with *L. braziliensis* from Corte de Pedra (the same endemic region we studied here) display low frequencies of neutrophils (26). This was confirmed by our data, showing that CD4+ and CD8+ T cells compose the great majority of cellular infiltrate present in the analyzed lesions. In addition, transcriptome studies from lesions of patients from Corte de Pedra showed a lack of neutrophil associated transcripts (43).

Our further analysis using anti-CD8, anti-CD107a and DAPI co-staining showed that the frequency of CD8+ T cells releasing LETs is clearly associated with the progression from the early to the late, ulcerated, phase of cutaneous leishmaniasis (CL), as well as with disease severity, since the lesions obtained from patients with the mutilating mucosal form (ML) also display more CD8-derived LETs. In addition, the percentage of CD8+ cells expressing the cytotoxic marker CD107a in etosis increased with the progression from early to late CL, as well as with the establishment of ML. Importantly, CD8+CD107+ cells releasing LETs were positively correlated with the intensity of the inflammatory infiltrate in late CL and in ML lesions, which shows a clear association of this process with progression and severity of local tissue pathology. The use of inflammatory infiltrate intensity as surrogate of pathology was chosen as it is not possible to measure the size lesions in early CL, as lesions are not yet ulcerated, and the measure of size of ML lesions is quite uncertain due to their location (mainly internal regions of nose and throat). These data emphasize the role of CD8 T cells in mediating tissue destruction in human leishmaniasis, suggesting that the release of LETs carrying cytotoxic vesicles by these cells may be one important mechanism by which CD8+ T cells induce tissue pathology. The use of molecules that inhibit the release of ETs has been proposed as therapeutic alternatives in inflammatory diseases were ETs play a role (63). Thus, it is possible that CD8-derived LETs may also be a therapeutic target in human TL, bettering the lives of millions.

Whether CD8+ T cell derived LETs induce pathology by amplifying inflammation or directly by causing death of tissue cells cannot be responded based on the tissue analysis we performed. In fact, these activities may not exclude one another. Yet, another possible function of CD8+ T cell-derived LETs is the elimination of *Leishmania*-infected cells. In addition, other cell types present in the lesions may also be able to release LETs and contribute to tissue destruction and/or parasite control. Finally, comparing the role of LET-associated cytotoxicity versus other classic mechanisms of cytotoxicity will bring valuable information regarding disease pathology. These are important points that, while not the focus of the current work, are under investigation in our lab.

Our results suggest that the release of LETs by CD8+ T cells may present a novel mechanism of cell-cell communication, likely associated with the delivery of CD107a+ cytotoxic vesicles to distant cells. These findings bring new insights to the understanding of CD8-mediated cytotoxicity and have critical implications in physiologic and pathologic mechanisms where CD8-induced cell death plays a critical role such as in infections by intracellular pathogens and cancer.

## Data Availability Statement

All datasets presented in this study are included in the article/**Supplementary Material**.

## Ethics Statement

The studies involving human participants were reviewed and approved by COEP—Federal University of Bahia. The patients/participants provided their written informed consent to participate in this study.

## Author Contributions

CK, AW, MV, LP, EN, TV, and EG performed the confocal, EM, FACS analysis. CK and AW also contributed to study design and analysis. PRLM and EC were responsible for care and material collection from leishmaniasis patients. DF performed the stainings and analysis of leishmania lesions. PMM overlooked all EM experiments and analysis. LA and RG contributed to the sorting experiments. GM contributed to study design and provided anti-histone antibodies. KG and WD designed the studies and overlooked all experiments and data analysis. All authors contributed to the article and approved the submitted version.

## Funding

This work was supported by FAPEMIG (#Universal 2014), CNPq (#Universal 2015), INCT-DT. CK, EG, LP, LA, GM, RG, PRLM, EC, KG, and WD are CNPq fellows and EGAN was a FAPEMIG fellow.

## Conflict of Interest

The authors declare that the research was conducted in the absence of any commercial or financial relationships that could be construed as a potential conflict of interest.

## Supplementary Material

Supplementary Figure 1 Anti-CD3/anti-CD28-stimulated cell cultures display low cell death. Cell death was assessed by annexin V and propidium iodide (Pi) staining using flow cytometry. Cultures treated with staurosporine were used as a positive control for apoptosis. **(A)** shows total cell death, **(B)** shows occurrence of early apoptosis and **(C)** shows frequency of necrosis. Data is presented as average per group ± standard error of the mean. The presence of the same letters in different bars represents statistically significant differences between them (p <0.05). Bottom panels show representative FACS plots of annexinV and PI staining for each condition.

Supplementary Figure 2 Lesions of patients with different forms of tegumentary leishmaniasis display extracellular DNA associated with tissue pathology. Frozen tissue sections were stained with DAPI, PE-labeled anti-CD8 and FITC-labeled anti-CD107a monoclonal antibodies as described in Materials and Methods. Inflammatory infiltrate, and frequency of CD8+ and CD8+CD107+ cells were determined as described in Material and Methods. **(A)** Inflammatory infiltrate in lesions of patients with the different clinical forms. Results are presented as boxplots with the median for all fields counted for each clinical form (Early-CL n=2, 27 fields; late-CL n=4, 43 fields; ML n=2, 28 fields). **(B)** Correlation analysis between the number of CD8+ cells and number of cells in the inflammatory infiltrate for each clinical form. **(C)** Correlation analysis between the number of CD8+CD107+ cells and number of cells in the inflammatory infiltrate for each clinical form.

Supplementary Video 1 Video showing 3D image of CD8+ T cells after stimulation with anti-CD3/anti-CD28. Purification of CD8 T lymphocytes was done by sorting, as described in Material and Methods. TCD8+ lymphocytes were stained with CFSE, plated on poly-L-lysine coverslips and stimulated with anti-CD3/anti-CD28 for 24 h. After incubation the cells were stained with DAPI as described in the Materials and Methods and analyzed in a confocal microscope.

Supplementary Video 2 Extracellular DNA from CD8+ T cells induce death of neighboring cells. Purified CD8 T cells were cultured with CFSE-labeled targets (pink) at a ratio of 1:4. Cultures were stimulated with anti-CD3/anti-CD28+ionomycin and stained with live-dead marker (EthD-1), as described in Materials and Methods. Images were obtained in 10-s intervals using excitation/emission captures of 495/515 nm for CFSE and 532/635 for EthD-1, on a Zeiss 5-live microscope. In (a), the movie shows the release of extracellular traps by a CD8+ T cell (light blue) and non-CD8 target cells stained in CFSE (pink) (arrow). Following, upon release of the LET, the pink cell dies after contact with the LET, becoming stained in light blue. In (b), the sequence of static frames, highlighting the box with the occurrence of etosis and death of the cell previously stained in pink (described in figure 3). In (c), there is an image of a cell in light blue (cell 1, CD8+ T cell stained with EthD-1) and one in pink (cell 2, target stained with CFSE), followed by intensity fluorescence histograms for each cell. The light blue curve represents the staining with EthD-1 and the pink curve represents the staining with CFSE. (d) Shows an image after the death of the pink cell by LETs. In addition, the profiles and the fluorescence intensities of EthD-1 (light blue) for the LETs are shown in the box. The video was recorded at a rate of 30 frames per second and corresponds from 14 h 16 min to 14 h 30 min of culture.
