# Supplementary figures and images for: Human CD8+ T Cells Release Extracellular Traps Co-Localized With Cytotoxic Vesicles That Are Associated With Lesion Progression and Severity in Human Leishmaniasis
Source: Front Immunol. 2020 Oct 8;11:594581. doi: 10.3389/fimmu.2020.594581 (PMC7578246; doi:10.3389/fimmu.2020.594581)

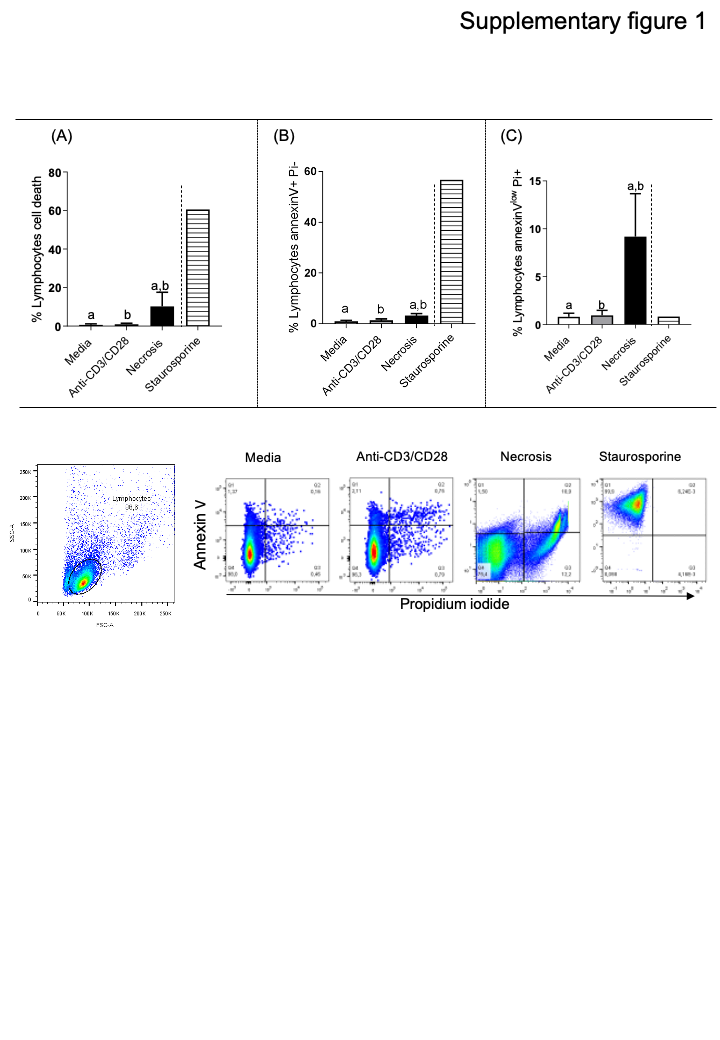

Supplement: Supplementary Figure 1 — Anti-CD3/anti-CD28-stimulated cell cultures display low cell death. Cell death was assessed by annexin V and propidium iodide (Pi) staining using flow cytometry. Cultures treated with staurosporine were used as a positive control for apoptosis. (A) shows total cell death, (B) shows occurrence of early apoptosis and (C) shows frequency of necrosis. Data is presented as average per group ± standard error of the mean. The presence of the same letters in different bars represents statistically significant differences between them (p <0.05). Bottom panels show representative FACS plots of annexinV and PI staining for each condition. [file Image_1.tiff]

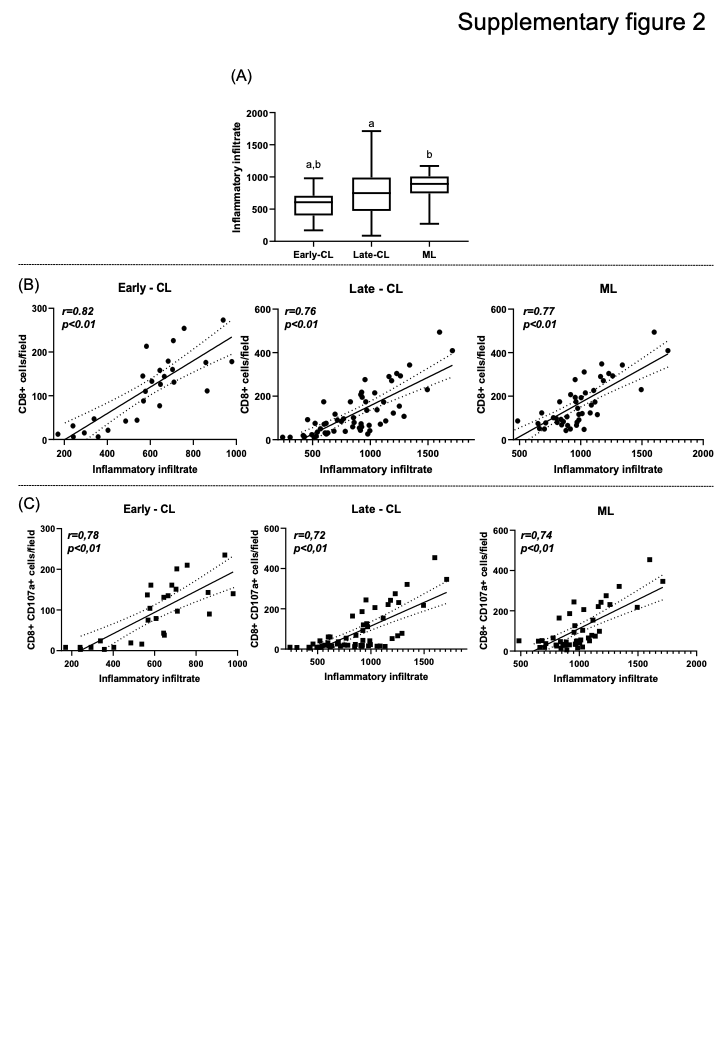

Supplement: Supplementary Figure 2 — Lesions of patients with different forms of tegumentary leishmaniasis display extracellular DNA associated with tissue pathology. Frozen tissue sections were stained with DAPI, PE-labeled anti-CD8 and FITC-labeled anti-CD107a monoclonal antibodies as described in Materials and Methods. Inflammatory infiltrate, and frequency of CD8+ and CD8+CD107+ cells were determined as described in Material and Methods. (A) Inflammatory infiltrate in lesions of patients with the different clinical forms. Results are presented as boxplots with the median for all fields counted for each clinical form (Early-CL n=2, 27 fields; late-CL n=4, 43 fields; ML n=2, 28 fields). (B) Correlation analysis between the number of CD8+ cells and number of cells in the inflammatory infiltrate for each clinical form. (C) Correlation analysis between the number of CD8+CD107+ cells and number of cells in the inflammatory infiltrate for each clinical form. [file Image_2.tiff]
